# Playing-Related Musculoskeletal Pain in Student and Professional Musicians: Associations with Practice Habits and Healthcare Support

**DOI:** 10.3390/ijerph23070937

**Published:** 2026-07-22

**Authors:** Cristina Schenone, Mehrnaz Hamedani, Emanuele Cambiaso, Giulia Mennella, Angelo Schenone, Valeria Prada

**Affiliations:** Department of Neurosciences, Rehabilitation, Ophthalmology, Genetics and Maternal/Child Sciences, University of Genova, 16132 Genova, Italy; crischenone92@gmail.com (C.S.); mehrnaz.hamedani@medicina.unige.it (M.H.); giumennella1994@libero.it (G.M.); aschenone@neurologia.unige.it (A.S.)

**Keywords:** musculoskeletal diseases, musicians, overuse injury, performing arts medicine, physiotherapy, health education

## Abstract

**Highlights:**

**Public health relevance—How does this work relate to a public health issue?**

- Playing-related musculoskeletal pain is highly prevalent among musicians, affecting approximately 75% of the sample studied, confirming that musculoskeletal disorders in musicians represent a relevant occupational and public health concern.
- The study confirms a high prevalence of playing-related musculoskeletal pain and shows that musicians reporting pain tended to report longer practice duration, although the corrected continuous-variable analyses did not reach statistical significance.

**Public health significance—Why is this work of significance to public health?**

- This work emphasizes the need for preventive strategies and early rehabilitation interventions in a population often overlooked by healthcare systems despite the high burden of musculoskeletal symptoms.
- The findings reveal a substantial gap between musicians and healthcare professionals, underlining the importance of improving access to specialized support and health education.

**Public health implications—What are the key implications or messages for practitioners, policy makers and/or researchers in public health?**

- Healthcare professionals and educational institutions should promote preventive programs focused on posture education, workload management, active breaks, and early physiotherapy interventions to reduce the risk of overuse-related musculoskeletal disorders in musicians.
- Future public health and research initiatives should strengthen collaboration between musicians, educational institutions, physiotherapists, and other healthcare providers.

**Abstract:**

Playing-related musculoskeletal pain is common among musicians and may affect performance, quality of life, and professional continuity. This exploratory cross-sectional study investigated the prevalence and characteristics of playing-related musculoskeletal pain in student and professional musicians. An anonymous survey was administered to student and professional musicians. Data were analyzed descriptively; normality was assessed using the Shapiro–Wilk test, and between-group comparisons were performed using Mann–Whitney U tests, chi-square tests, or Fisher exact tests as appropriate. Effect sizes and 95% confidence intervals were reported where possible. Ninety-seven musicians participated, including 61 students and 36 professionals. Seventy-three participants (75.3%) reported pain while playing. Compared with musicians without pain, musicians reporting pain showed numerically higher daily practice duration, weekly practice duration, and uninterrupted practice duration, but these differences were not statistically significant. Only 31 participants (32.0%) reported having consulted a physiotherapist. The associations between practice duration and pain were weak and not statistically significant after the corrected analysis. Owing to the cross-sectional design and self-reported outcomes, these findings should be interpreted cautiously and not as evidence of causality. Preventive education, workload management, and improved access to healthcare professionals with expertise in performing arts medicine may help reduce the burden of musculoskeletal symptoms in musicians.

## 1. Introduction

Playing-related musculoskeletal disorders (PRMDs) include pain, numbness, weakness, tingling, loss of flexibility, or other musculoskeletal symptoms that interfere with the ability to play an instrument [1]. The conditions most often diagnosed in musicians include tendinitis, bursitis, tenosynovitis, focal dystonia, and entrapment neuropathies [2].

The prevalence of musculoskeletal pain or playing-related musculoskeletal disorders among musicians has been reported to range widely, from approximately 64% to 85% in several earlier studies [3,4,5,6,7]. This variability likely reflects differences in study populations, definitions of playing-related symptoms, instruments played, and assessment methods. More recent systematic reviews and multicenter studies have further emphasized the high burden of musculoskeletal pain among musicians and the importance of validated, musician-specific assessment tools [8,9,10,11,12,13,14]. The prevalence of pain can vary greatly in relation to the different samples of musicians studied in various studies as well as in respect of age, sex and instrument played by the candidates. The instrument played assumes a pivotal role as a risk factor in the development of musculoskeletal disorders, especially for string instrumentalists and pianists [7,15,16]. These two categories have shown a higher prevalence of the pain in the upper limbs, shoulders and neck areas [17,18,19]. However, it is important to notice that significant differences can be observed in players among the same class of instruments and while violinists are more frequently affected in the upper limbs, neck and temporomandibular joint areas [19], cellists more commonly report impairment in the shoulders and upper back areas [20]. In pianists, symptoms are usually found in a similar location to that of string instrumentalists, albeit with higher prevalence rates in the hands and forearms [21].

The current literature suggests that musculoskeletal symptoms in musicians often develop over time and may begin early in advanced musical training. Several studies have reported that 25% to 43% of university-level music students had already experienced pain before entering university or during the first years of university-level education [22,23]. This period may be particularly demanding because students often experience a steep increase in practice hours when moving from pre-academic to academic musical training [24]. Associations between practice duration and pain have raised concern about overuse-related symptoms, which have been described as painful conditions associated with prolonged or excessive use of a limb in relation to the individual’s tolerance [7,25].

Healthcare professionals can support musicians through education on risk factors, advice on practice planning and workload management, early diagnosis and treatment, and appropriate post-injury support [26]. Careful organization of musical practice may decrease injury risk by allowing adequate recovery while maintaining technical development [27].

The aim of this exploratory study was to investigate the prevalence and characteristics of pain in student and professional musicians, to examine whether pain was associated with practice habits, with particular attention to uninterrupted practice duration, and to explore whether musicians were adequately followed by healthcare professionals. We hypothesized that musicians reporting pain would have longer uninterrupted practice sessions than those without pain and that healthcare engagement, particularly physiotherapy consultation, would be limited.

## 2. Materials and Methods

Recruitment. This study was conducted in collaboration with the Conservatorio Niccolò Paganini of Genoa and the Conservatorio Antonio Vivaldi of Alessandria. The sample included both students and professional musicians affiliated with the participating institutions, without restrictions based on instrument type. This approach allowed us to describe a broad and heterogeneous population of musicians.

Participation was voluntary and the only inclusion criterion was being a student or professional musician affiliated with one of the participating Conservatories. No a priori sample-size calculation was performed because the study was designed as an exploratory survey and recruitment depended on voluntary participation.

Survey. An anonymous survey in Italian language was developed by the research team to collect information on demographic characteristics, musical practice, lifestyle, pain during musical activity, pain impact, and healthcare support. The questionnaire included 37 questions, of which 34 were closed-ended and 3 were open-ended, divided into 4 sections: general characteristics, musical practice experience, hobbies and daily life, and pain management. The questionnaire collected categorical responses and numerical information on practice duration; no global score was calculated. The survey was not formally validated and no psychometric reliability testing was performed; therefore, it should be considered an exploratory data-collection tool. This limitation was taken into account when interpreting the findings. The full list of items and response options is provided as Supplementary File S1 to improve reproducibility. The questionnaire was administered in person at the schools from April 2019 to November 2019, together with an informed consent form. Signed informed consent forms were separated from the questionnaires to maintain anonymity. Participants were enrolled in a larger study approved by the CER Liguria Ethics Committee (approval 1170/2018). For underage participants, written consent was obtained from parents or legal guardians. To increase professional participation, the questionnaire was also transposed into digital format and sent by e-mail using the public addresses available on the conservatories’ websites. The data were then extracted in Microsoft Excel for preliminary analysis.

Statistical analyses. Data were entered in Microsoft Excel, checked for consistency, and analyzed after linking blank continuation rows to the corresponding participant identifier for multi-response variables. Participant-level analyses were based on the first complete row for each participant, whereas multi-response variables such as pain regions, specialists, and treatments were summarized as multiple mentions. Continuous variables were reported as mean ± standard deviation and, where relevant, as median [interquartile range]. Categorical variables were reported as counts and percentages. Normality was assessed using the Shapiro–Wilk test. Because several variables were not normally distributed and subgroup sizes were unequal, between-group comparisons for continuous variables were performed using the Mann–Whitney U test. The Mann–Whitney U test was used as a non-parametric alternative to the independent-samples t-test and was not considered a post-test. Categorical variables were compared using chi-square tests or Fisher exact tests when appropriate. Statistical significance was set at *p* < 0.05. For the main prevalence estimates, 95% confidence intervals were calculated using the Wilson method. Rank-biserial correlation was reported as an effect-size estimate for Mann–Whitney U tests. Because the study was exploratory, *p*-values were not adjusted for multiple comparisons.

## 3. Results

Generalities. Ninety-seven musicians participated in the study. The study population included 59 males (60.8%) and 38 females (39.2%); 61 participants were students (62.9%) and 36 were professionals (37.1%). The mean age of the whole sample was 34.6 ± 17.2 years (range 12–65). The student group included 35 males (57.4%) and 26 females (42.6%), with a mean age of 22.0 ± 3.3 years (range 12–32). The professional group included 24 males (66.7%) and 12 females (33.3%), with a mean age of 55.9 ± 6.7 years (range 34–65). The mean total duration of musical practice was 24.5 ± 18.2 years for the entire sample, 11.3 ± 3.9 years for students, and 47.0 ± 7.4 years for professionals. The mean age at the beginning of musical practice was 9.8 ± 3.5 years (Table 1).

**Figure 1:**
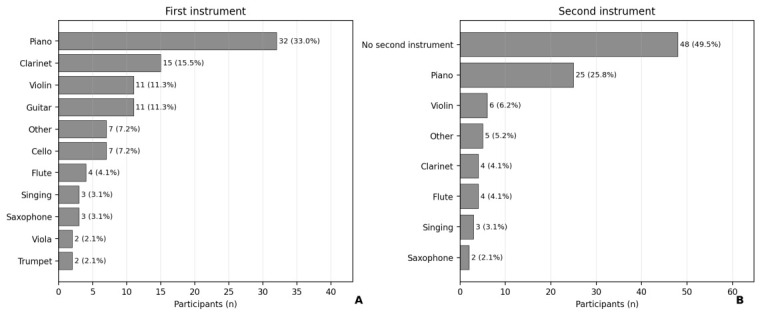
Instruments played by the participating musicians (*n* = 97). (**A**) First instrument played. Piano was the most frequent first instrument (33.0%; *n* = 32), followed by clarinet (15.5%; *n* = 15), guitar (11.3%; *n* = 11), violin (11.3%; *n* = 11), cello (7.2%; *n* = 7), flute (4.1%; *n* = 4), singing (3.1%; *n* = 3), saxophone (3.1%; *n* = 3), trumpet (2.1%; *n* = 2), viola (2.1%; *n* = 2), and other less represented instruments. (**B**) Second instrument played. Nearly half of participants did not play a second instrument (49.5%; *n* = 48). Piano was the most frequent second instrument (25.8%; *n* = 25), followed by violin (6.2%; *n* = 6), clarinet (4.1%; *n* = 4), flute (4.1%; *n* = 4), singing (3.1%; *n* = 3), saxophone (2.1%; *n* = 2), and other instruments.

The most frequently represented first instruments were piano (33.0%; *n* = 32), clarinet (15.5%; *n* = 15), guitar (11.3%; *n* = 11), violin (11.3%; *n* = 11), cello (7.2%; *n* = 7), flute (4.1%; *n* = 4), saxophone (3.1%; *n* = 3), singing (3.1%; *n* = 3), trumpet (2.1%; *n* = 2), and viola (2.1%; *n* = 2). Seven participants played other first instruments. A second instrument was reported by 49 participants (50.5%). The most frequent second instruments were piano (25.8%; *n* = 25), violin (6.2%; *n* = 6), flute (4.1%; *n* = 4), clarinet (4.1%; *n* = 4), singing (3.1%; *n* = 3), and saxophone (2.1%; *n* = 2), while 48 participants (49.5%) reported no second instrument (Figure 1).

**Figure 2:**
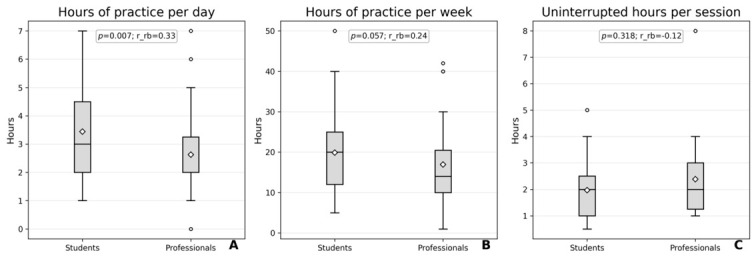
Differences between students and professionals in daily, weekly, and uninterrupted practice duration. Boxplots show median, interquartile range, and individual outliers; diamonds indicate means. (**A**) Students reported significantly more daily practice than professionals (students: 3.4 ± 1.5 h; professionals: 2.6 ± 1.5 h; U = 1416.0; *p* = 0.007; rank-biserial r = 0.33). (**B**) Weekly practice duration did not differ significantly between groups (students: 19.9 ± 9.1 h; professionals: 16.9 ± 10.8 h; U = 1275.5; *p* = 0.057; rank-biserial r = 0.24). (**C**) Uninterrupted practice duration was not significantly different between groups (students: 2.0 ± 1.0 h; professionals: 2.4 ± 1.7 h; U = 907.5; *p* = 0.318; rank-biserial r = −0.12).

Musical Practice. The hours of daily, weekly, and uninterrupted practice per session were calculated for the entire sample and separately for the student and professional groups. For the entire sample, mean daily practice was 3.1 ± 1.5 h (*n* = 96), mean weekly practice was 18.8 ± 9.8 h (*n* = 94), and mean uninterrupted practice per session was 2.1 ± 1.3 h (*n* = 94). Students reported significantly more daily practice than professionals (students: 3.4 ± 1.5 h; professionals: 2.6 ± 1.5 h; U = 1416.0; *p* = 0.007; rank-biserial r = 0.33, Figure 2A). Weekly practice duration showed a non-significant trend toward higher values in students (students: 19.9 ± 9.1 h; professionals: 16.9 ± 10.8 h; U = 1275.5; *p* = 0.057; rank-biserial r = 0.24, Figure 2B). Uninterrupted practice duration did not differ significantly between students and professionals (students: 2.0 ± 1.0 h; professionals: 2.4 ± 1.7 h; U = 907.5; *p* = 0.318; rank-biserial r = −0.12, Figure 2C).

**Figure 3:**
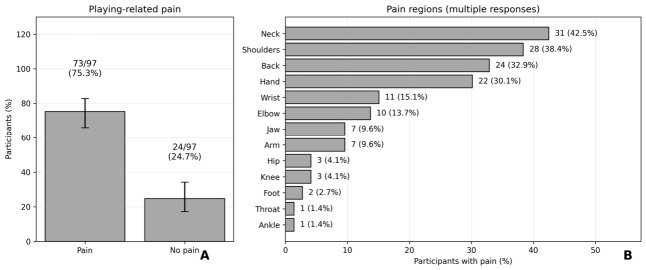
Presence of pain and anatomical regions affected by pain in musicians. (**A**) Seventy-three participants (75.3%; 95% CI 65.8–82.8) reported having experienced pain while playing, while 24 (24.7%) did not report playing-related pain. (**B**) Pain regions among participants reporting playing-related pain. Multiple responses were allowed; therefore, percentages are calculated over the 73 participants with pain and do not sum to 100%. The most frequently affected regions were the neck (*n* = 31), shoulders (*n* = 28), back (*n* = 24), and hands (*n* = 22). Other reported regions included the wrists (*n* = 11), elbow (*n* = 10), mandible (*n* = 7), arms (*n* = 7), hip (*n* = 3), knee (*n* = 3), foot (*n* = 2), throat (*n* = 1), and ankle (*n* = 1).

Pain. Seventy-three participants (75.3%; 95% CI 65.8–82.8) answered affirmatively when asked whether they had ever experienced pain while playing (Figure 3A). A similar percentage of students reported pain (78.7%; *n* = 48/61), while 69.4% of professionals reported pain while playing in the past (*n* = 25/36); this difference was not statistically significant (Fisher exact test, *p* = 0.337). Pain occurrence was comparable in females and males, with affirmative answers reported by 76.3% of females (*n* = 29/38) and 74.6% of males (*n* = 44/59; Fisher exact test, *p* = 1.000). Sixteen participants (16.5%; 95% CI 10.4–25.1) reported pain at the time of the questionnaire. Current pain was reported by 16.4% of students (*n* = 10/61), 16.7% of professionals (*n* = 6/36), 11.9% of males (*n* = 7/59), and 23.7% of females (*n* = 9/38). Among participants with pain, the most frequently reported self-perceived causes were posture (*n* = 21), overuse (*n* = 14), incorrect technique (*n* = 4), muscular tension (*n* = 3), arthrosis (*n* = 3), and challenging repertoire (*n* = 2). Because multiple anatomical regions could be reported, percentages do not sum to 100%. The most frequently affected anatomical areas were the neck (*n* = 31; 42.5% of participants with pain), shoulders (*n* = 28; 38.4%), back (*n* = 24; 32.9%), and hands (*n* = 22; 30.1%), followed by the wrists, elbow, mandible, and arm (Figure 3B).

**Figure 4:**
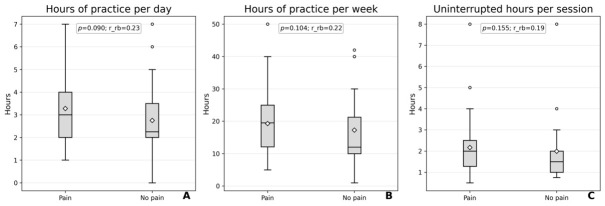
Association between pain and practice behavior. Boxplots show median, interquartile range, and individual outliers; diamonds indicate means. (**A**) Daily practice duration did not differ significantly between musicians with and without pain (pain group: 3.28 ± 1.47 h; no-pain group: 2.75 ± 1.60 h; U = 1063.0; *p* = 0.090; rank-biserial r = 0.23). (**B**) Weekly practice duration did not differ significantly between groups (pain group: 19.31 ± 8.88 h; no-pain group: 17.27 ± 12.22 h; U = 1027.5; *p* = 0.104; rank-biserial r = 0.22). (**C**) Uninterrupted playing duration was numerically higher in musicians with pain but was not statistically significant (pain group: 2.17 ± 1.21 h; no-pain group: 1.99 ± 1.57 h; U = 975.0; *p* = 0.155; rank-biserial r = 0.19).

Pain in relation to musical practice. We then examined the association between pain and practice habits. Daily practice duration did not differ significantly between musicians with and without pain (*n* = 96; pain group: 3.28 ± 1.47 h, median 3.0 [2.0–4.0]; no-pain group: 2.75 ± 1.60 h, median 2.25 [2.0–3.5]; U = 1063.0; *p* = 0.090; rank-biserial r = 0.23; Figure 4A). Weekly practice duration also did not differ significantly between groups (*n* = 94; pain group: 19.31 ± 8.88 h, median 19.5 [12.1–25.0]; no-pain group: 17.27 ± 12.22 h, median 12.0 [10.0–21.3]; U = 1027.5; *p* = 0.104; rank-biserial r = 0.22; Figure 4B). Uninterrupted playing duration was numerically higher in musicians reporting pain, but the difference was not statistically significant after the corrected analysis (*n* = 94; pain group: 2.17 ± 1.21 h, median 2.0 [1.3–2.5]; no-pain group: 1.99 ± 1.57 h, median 1.5 [1.0–2.0]; U = 975.0; *p* = 0.155; rank-biserial r = 0.19; Figure 4C). These results indicate only weak, non-significant associations between practice duration and pain occurrence and do not establish causality.

We found no significant relationships between pain and age (U = 864.0; *p* = 0.923; rank-biserial r = −0.01), age at the beginning of musical practice (U = 992.0; *p* = 0.332; rank-biserial r = 0.13), or total years of musical practice (U = 828.0; *p* = 0.691; rank-biserial r = −0.05) (Supplementary Material).

We investigated the relationship between athleticism and pain. Forty-nine participants (50.5%) reported practicing at least one sport, while 48 (49.5%) did not. Pain was reported by 35 of 49 athletic participants (71.4%) and by 38 of 48 non-athletic participants (79.2%). The association between athleticism and pain was not statistically significant (Fisher exact test, *p* = 0.482).

**Figure 5:**
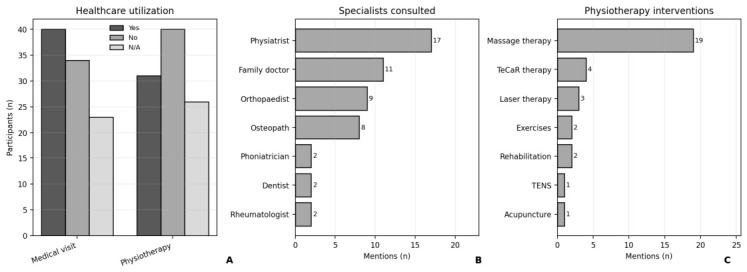
Healthcare support and treatment among participants. (**A**) Healthcare utilization, including medical visits and physiotherapy consultation, reported as yes/no/not applicable. (**B**) Types of healthcare professionals consulted; multiple responses were possible. (**C**) Types of physiotherapeutic interventions reported; multiple responses were possible. Massage therapy was the most frequently reported intervention.

Medical support and therapy. Forty participants (41.2%; 95% CI 32.0–51.2) reported having sought medical consultation for their symptoms, 34 (35.1%) reported that they had not, and 23 (23.7%) did not provide an applicable answer (Figure 5A). Among participants reporting playing-related pain, 36 of 73 (49.3%) had sought medical consultation and 28 of 73 (38.4%) had consulted a physiotherapist. In the whole sample, 31 participants (32.0%; 95% CI 23.5–41.8) reported having consulted a physiotherapist (Figure 5B). The most frequently consulted specialists were physiatrists (*n* = 17), family doctors (*n* = 11), orthopaedists (*n* = 9), and osteopaths (*n* = 8). The most frequently reported physiotherapeutic intervention was massage therapy/massotherapy (*n* = 19), followed by TeCaR therapy (*n* = 4), laser therapy (*n* = 3), exercises (*n* = 2), and rehabilitation treatment (*n* = 2; Figure 5C).

## 4. Discussion

This study showed that playing-related musculoskeletal pain was highly prevalent among student and professional musicians. After reanalysis of the complete dataset using the specified Mann–Whitney U tests and participant-level data, practice-duration variables showed only weak, non-significant associations with pain occurrence. Therefore, these findings should be interpreted as exploratory trends rather than evidence that longer practice duration causes pain. The results also point to a relevant gap between musicians and healthcare professionals, since only a minority of participants reported physiotherapy consultation despite the high prevalence of symptoms.

Students represented 63% of the sample and included candidates at different stages of musical education, from early training to more advanced academic and professional development. Professionals represented 37% of the sample. This imbalance between groups was expected, given the usual ratio between professors and students in conservatory settings. Regarding sex distribution, males represented 61% of the sample and females 39%, which differs from several previous studies in which females were more frequently represented [15,28,29].

Similarly, to what was previously stated about age and professional level, our sample was highly heterogeneous in terms of instrument played and musical background. This heterogeneity increases the ecological relevance of the study but limits the possibility of identifying instrument-specific risk profiles. In response to this issue, we performed an exploratory instrument-group analysis. Pain prevalence was 78.1% among pianists, 80.0% among bowed-string players, 54.5% among guitarists, 73.1% among wind players, and 87.5% among voice/other instrumentalists. However, the association between instrument group and pain was not statistically significant (chi-square test, *p* = 0.459), and the small cell sizes limit interpretation. Potential confounding by instrument type therefore remains possible and should be further examined in larger studies.

Professionals and students had broadly similar practice habits in terms of weekly and uninterrupted practice duration, although students reported more hours of daily practice. This difference may reflect additional individual practice performed by students outside formal lessons and classes.

Pain was present in most musicians, without a clear distinction between students and professionals. In the corrected continuous-variable analyses, daily, weekly, and uninterrupted practice duration were numerically higher in musicians with pain, but none of these comparisons reached statistical significance. This finding suggests that the organization of practice time may still be clinically relevant, but the present data do not provide robust statistical evidence for an association. Alternative explanations are possible: musicians with pain may modify their practice habits, musicians with higher workloads may be more exposed to pain, and unmeasured factors such as instrument type, technique, psychological stress, or previous injuries may influence both practice duration and pain occurrence. Therefore, the present data cannot establish a causal relationship between uninterrupted practice and pain.

No significant association was found between pain and total years of musical practice, professional career duration, or age at the beginning of musical practice. These findings suggest that, in this sample, pain was not simply related to cumulative years of exposure. Other factors, such as practice organization, posture, technique, individual predisposition, instrument-specific demands, or psychosocial factors, may therefore be relevant and should be explored in future studies.

Regarding pain characteristics, our findings were broadly consistent with previous literature with respect to the most affected anatomical areas and instrument groups. The neck, shoulders, back, and hands were the most frequently reported pain regions, in line with the high mechanical demands placed on the upper limb, cervical spine, and shoulder girdle during musical performance. Because participants could report multiple pain regions, the anatomical distribution should be interpreted as the frequency of reported regions rather than mutually exclusive diagnoses.

The last part of our survey aimed to analyze the relationship between musicians and healthcare professionals. Despite the high prevalence of pain observed in this sample, only 41.2% of the whole sample reported having sought medical consultation and only 32.0% reported having consulted a physiotherapist; even among participants with pain, physiotherapy consultation was reported by only 38.4%. This apparent disconnect may reflect multiple barriers, including limited awareness of performing arts medicine services, the tendency of musicians to normalize pain as part of training, concerns about career consequences, limited access to specialized healthcare providers, and insufficient communication between conservatories and healthcare services. Recent multicenter studies using validated tools have similarly highlighted that performance-related pain is often under-recognized and that a relevant proportion of musicians do not seek healthcare despite pain and disability [11,12,13]. These findings support the need to improve prevention, early referral pathways, and interdisciplinary collaboration between musicians, teachers, physiotherapists, physicians, and performing arts medicine specialists.

Therapeutic treatments appeared to be limited and heterogeneous. In our study, only a subset of participants reported receiving physiotherapeutic treatment, and the most frequently reported intervention was described generically as “massage therapy”. This terminology is non-specific and may include different manual techniques; however, it may also indicate treatment without a precise diagnosis or structured rehabilitation plan. More specific interventions such as TeCaR therapy, laser therapy, iontophoresis, or rehabilitation treatment were reported by a lower proportion of participants. These findings suggest that musicians may benefit from more standardized clinical assessment, education, and individualized rehabilitation programs addressing posture, workload distribution, active breaks, strength, mobility, and instrument-specific demands.

Several limitations should be considered. First, the questionnaire was developed by the authors and was not formally validated; no reliability, validity, or reproducibility testing was performed. Although the questionnaire allowed broad exploratory data collection, the absence of a validated instrument limits comparability with other studies and may reduce measurement robustness. Future studies should consider using validated musician-specific instruments, such as the Performance-related Pain Among Musicians Questionnaire or the Musculoskeletal Pain Intensity and Interference Questionnaire for Musicians [9,10,11].

Second, the anonymous self-reported survey design facilitated participation and may have encouraged musicians to report health problems freely, but it also prevented verification of clinical diagnoses and detailed follow-up of individual symptom patterns. Pain location, causes, and treatment history were based on participants’ perceptions and were not confirmed through medical examination. Third, the cross-sectional design does not allow causal inference between practice duration and pain. Fourth, no a priori power analysis was performed, and the relatively small and heterogeneous sample limits generalizability, particularly for instrument-specific analyses. Fifth, the corrected analysis showed that the association between uninterrupted practice duration and pain was not statistically significant, meaning that this finding should be considered hypothesis-generating rather than confirmatory.

Moreover, we did not investigate specific diagnoses in depth and we cannot determine whether each reported symptom was directly caused by musical practice. We can only report the prevalence of self-reported pain and exploratory, non-causal associations with practice characteristics. Future research should include larger and more homogeneous samples, validated questionnaires, instrument-specific analyses, and, where possible, objective clinical assessment to better characterize diagnoses, risk factors, and treatment needs.

In addition, it would be important to further investigate the relationship between musicians and healthcare professionals, aiming to improve diagnostic and therapeutic pathways for this category of patients, whose symptoms are often underestimated and underdiagnosed. Conservatories and music institutions may play a key role by promoting health education, screening, access to physiotherapy, and early referral to clinicians familiar with the specific demands of musical performance.

## 5. Conclusions

In conclusion, playing-related musculoskeletal pain was highly prevalent among both student and professional musicians in this exploratory sample. After correction of the statistical analysis using the complete dataset, practice-duration variables were numerically higher in musicians with pain but were not statistically significant, whereas total years of musical experience and age were not significantly related to pain. Because of the cross-sectional design, self-reported outcomes, and limited sample size, these findings should be interpreted cautiously and do not demonstrate that prolonged practice directly causes pain. Nevertheless, the results support the practical relevance of workload distribution, structured active breaks, posture education, and early access to physiotherapy as preventive strategies. The low proportion of participants reporting medical or physiotherapeutic support highlights the need for stronger collaboration between musicians, conservatories, and healthcare professionals. Future studies should use validated assessment tools, include larger and more homogeneous samples, examine instrument-specific risk profiles, and evaluate targeted rehabilitation approaches.

## Figures and Tables

**Table 1:** Generalities of participants.

Samples (*n*)	97
Males/Females (*n*)	59/38
Females (%)	39.2
Average age, whole sample (years)	34.6 ± 17.2 (range 12–65)
Students/professionals (*n*)	61/36
Professionals (%)	37.1
Students average age (years)	22.0 ± 3.3 (range 12–32)
Professionals average age (years)	55.9 ± 6.7 (range 34–65)
Students’ years of practice	11.3 ± 3.9
Professionals’ years of practice	47.0 ± 7.4

## Data Availability

The data presented in this study are available on request from the corresponding author.

## Supplementary Materials

The following supporting information can be downloaded at: https://www.mdpi.com/article/10.3390/ijerph23070937/s1, Figure S1: Between-group comparisons for pain-related variables not shown in the main figures. Boxplots show median and interquartile range, whiskers indicate data spread, and diamonds indicate means; File S1: Questionnaire items and response options; Table S1: Statistical details for Figure S1.
