# Supplementary material for: Playing-Related Musculoskeletal Pain in Student and Professional Musicians: Associations with Practice Habits and Healthcare Support
Source: Int J Environ Res Public Health. 2026 Jul 22;23(7):937. doi: 10.3390/ijerph23070937 (PMC13411696; doi:10.3390/ijerph23070937)
Supplement: Supplementary file 1 [file ijerph-23-00937-s001.zip › ijerph-4364022 - Supplementary Materials - file S1.pdf]

Supplementary Material – Survey

The following author-developed questionnaire was administered in Italian and is reported here as an English translation for transparency and reproducibility. The questionnaire was used for exploratory purposes and was not formally validated.

General information and musical practice

- Age
- Sex: Male/Female
- How many years have you been playing your instrument?
- Role: Student/Teacher
- Please describe your experience as a musician.
- First instrument
- Second instrument
- How many hours per day do you practice?
- How many hours per week do you practice?
- How many hours do you usually play continuously in one session?
- At what age did you start studying music?

Hobbies and daily life

- Do you practice any sports? Yes/No. If yes, which one(s)?
- Do you practice any hobbies? Yes/No. If yes, which one(s)?
- Are you studying, or have you studied, anything else? Yes/No. If yes, what?
- Do you have a second job besides playing? Yes/No. If yes, what?
- Is playing fundamental for you? Yes/No
- Would you ever give up playing your instrument? Yes/No

Health issues and pain

- Have you ever had pain while playing in the past? Yes/No
- Have you had pain while playing in the last month? Yes/No
- Do you have pain while playing right now? Yes/No
- How long have you been experiencing pain?
- Is the pain constant or does it change over time?
- If it changes over time, what do you think it is connected to?
- Does the pain prevent you from completing your practice? Yes/No
- Does the pain prevent you from performing concerts? Yes/No
- Does the pain prevent you from performing at your usual level? Yes/No
- Does the pain prevent you from being happy and/or satisfied with your life? Yes/No
- Do you take medication to control pain? Yes/No/I have no pain
- If you have pain, where is it located? Neck; shoulder; elbow; wrist; mandible/jaw; hand; back (thoracic/lumbar); hip; knee; ankle; foot
- Do you think the pain is connected with your musical practice? Yes/No

Healthcare support and treatment

- Have you had any medical examinations for this problem? Yes/No
- If yes, which specialist did you consult? General practitioner; orthopaedist; physiatrist; rheumatologist; neurologist; other
- If other, which medical specialist?
- Have you ever been treated by a physiotherapist? Yes/No
- What physiotherapy treatments did you receive?
- Have you received a diagnosis for your problem? Yes/No

- Were you able to resolve your problem? Yes/No
- If you have not resolved the problem, are you currently undergoing therapy? Yes/No
- If you have not resolved the problem and are not currently undergoing therapy, how do you intend to proceed?

Table S1. Statistical details for Figure S1

Panel	Variable	Pain group	No-pain group	U	p-value	Effect size
A	Age at beginning of musical practice	n=73; mean±SD 10.05±3.62; median [IQR] 10.0 [8.0–12.0]	n=24; mean±SD 9.21±3.22; median [IQR] 9.5 [6.8–11.0]	992	0.332	rank-biserial r=0.13
B	Age	n=73; mean±SD 34.04±16.78; median [IQR] 24.0 [22.0–53.0]	n=24; mean±SD 36.21±18.52; median [IQR] 25.0 [21.0–55.0]	864	0.923	rank-biserial r=-0.01
C	Years of musical practice	n=73; mean±SD 23.74±17.66; median [IQR] 14.0 [11.0–40.0]	n=24; mean±SD 26.96±19.84; median [IQR] 15.0 [9.2–45.8]	828	0.691	rank-biserial r=-0.05
