# Supplementary material for: Playing-Related Musculoskeletal Pain in Student and Professional Musicians: Associations with Practice Habits and Healthcare Support
Source: Int J Environ Res Public Health. 2026 Jul 22;23(7):937. doi: 10.3390/ijerph23070937 (PMC13411696; doi:10.3390/ijerph23070937)
Supplement: Supplementary file 1 [file ijerph-23-00937-s001.zip › ijerph-4364022 - Supplementary Materials Figure S1.pdf]

Supplementary Figure S1

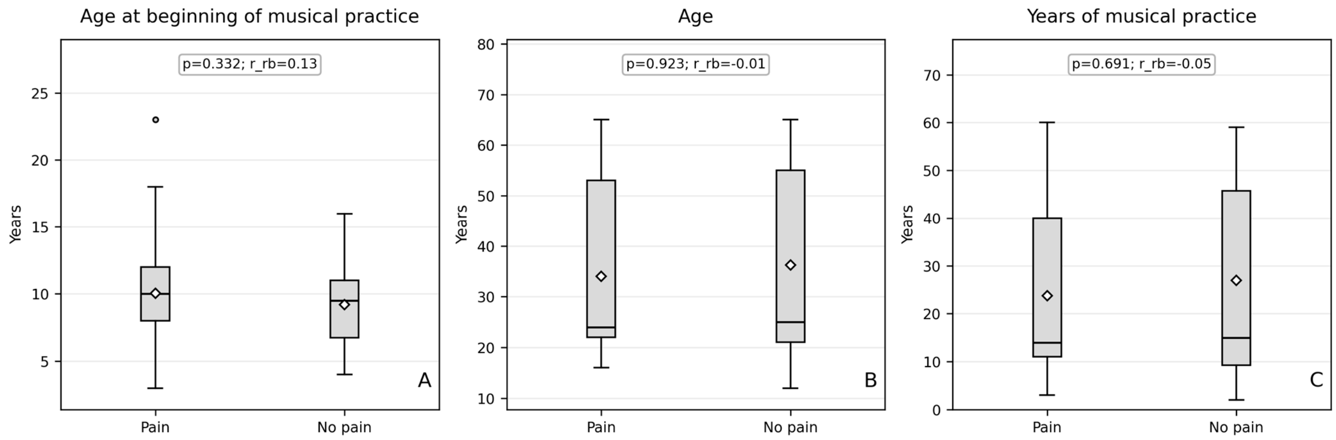

Figure S1. Between-group comparisons for pain-related variables not shown in the main figures. Boxplots show median and interquartile range, whiskers indicate data spread, and diamonds indicate means. A) Age at beginning of musical practice: Pain group, n=73, mean±SD 10.05±3.62, median [IQR] 10.0 [8.0–12.0]; No-pain group, n=24, mean±SD 9.21±3.22, median [IQR] 9.5 [6.8–11.0]; Mann–Whitney U=992, p=0.332, rank-biserial correlation=0.13. B) Age: Pain group, n=73, mean±SD 34.04±16.78, median [IQR] 24.0 [22.0–53.0]; No-pain group, n=24, mean±SD 36.21±18.52, median [IQR] 25.0 [21.0–55.0]; Mann–Whitney U=864, p=0.923, rank-biserial correlation=-0.01. C) Years of musical practice: Pain group, n=73, mean±SD 23.74±17.66, median [IQR] 14.0 [11.0–40.0]; No-pain group, n=24, mean±SD 26.96±19.84, median [IQR] 15.0 [9.2–45.8]; Mann–Whitney U=828, p=0.691, rank-biserial correlation=-0.05.
